# Gene expression, immunohistochemical and microarchitectural evaluation of bone formation around two implant surfaces placed in bone defects filled or not with bone substitute material

**DOI:** 10.1186/s40729-020-00279-7

**Published:** 2020-12-01

**Authors:** Guilherme dos Santos Trento, Jaqueline Suemi Hassumi, Paula Buzo Frigério, Ana Paula Farnezi Bassi, Roberta Okamoto, Marisa Aparecida Cabrini Gabrielli, Valfrido Antonio Pereira-Filho

**Affiliations:** 1 Department of Diagnosis and Surgery, School of Dentistry, Sao Paulo State University (Unesp), 1680th Humaitá Street, Araraquara, SP 14801-903 Brazil; 2 Department of Oral and Maxillofacial Surgery, School of Dentistry, São Paulo State University (Unesp), Araçatuba, Brazil

**Keywords:** Osseointegrated dental implantation, Bone grafting, Cell viability

## Abstract

**Objective:**

The aim of this study is to evaluate through gene expression, immunohistochemical and microtomographic (micro-CT) analysis the response of peri-implant bone tissue around titanium implants with different surface treatments, placed in bone defects filled or not with bone substitute materials. In addition, to investigate the hypothesis that porous-hydrophilic surface induces a faster bone formation.

**Materials and methods:**

Twenty-six animals were divided into two groups according to implant surface treatment. In each tibia, a bone defect was created followed by the placement of one implant. On the left tibia, the defect was filled with blood clot (BC), and on the right tibia, the defect was filled with biphasic hydroxyapatite/β-tricalcium-phosphate (HA/TCP) generating four subgroups: BC-N: bone defect filled with blood clot and porous surface titanium implant installed; BC-A: bone defect filled with blood clot and porous-hydrophilic surface titanium implant installed; HA/TCP-N: bone defect filled with bone substitute material and porous surface titanium implant installed; and HA/TCP-A: bone defect filled with bone substitute material and porous-hydrophilic surface titanium implant installed. The animals were submitted to euthanasia at 15, 30, and 60 days after implant installation. The expression of two genes was evaluated: RUNX2 and BSP. Immunohistochemical analyses were performed for detection of RUNX2, OPN, OCN, OPG, and RANKL antibodies and bone matrix proteins. Finally, four parameters were chosen for micro-CT analysis: trabecular number, separation and thickness, and connectivity density.

**Results:**

Descriptive analysis showed similar findings among the experimental groups. Moreover, porous-hydrophilic surfaces presented a higher expression of RUNX2, which is probably an indicative of better osteogenesis; although the data from this study may be considered an insufficient support for a concrete statement.

**Conclusion:**

Porous hydrophilic surface can improve and accelerate protein expression and bone formation.

## Introduction

Currently, the use of osseointegrated titanium implants for oral rehabilitation of partially or fully edentulous patients is a reliable therapy with high success rates [1, 2]. The integrity of bone-implant contact is an important factor that dictates the success of dental implant rehabilitation. After implant installation, several biological reactions, dependent by numerous factors, occur for bone repair [3]. Implant design, physico-chemical implant surface modifications, and the presence of bone substitute materials may influence those events [4, 5].

Some techniques have been reported in order to reduce the time for oral rehabilitation, such as immediate placement of dental implant in fresh extraction sockets. However, the socket can be considered a bone defect and a gap may exist between implant and remaining alveolar bone after placement [6, 7]. According to Quirynen et al. [8], the immediate dental implant installation generates some conditions that may or may not require guided bone regeneration (GBR).

Due to its biological properties of osteogenesis, osteoinduction, and osteoconduction, the autogenous bone graft is still considered as gold standard for bone grafting procedures. However, the harvesting of the graft is not always possible and bone substitute material may be necessary for adequate GBR [9, 10]. For that task, some bone substitute materials with strictly osteoconductive properties have been used. Biphasic ceramic composed of hydroxyapatite/β-tricalcium phosphate is one of them and its importance has been described due to high predictability in bone gain and longevity in oral rehabilitation [9, 11, 12].

Also, as a contributor for a faster osseointegration process, modifications of the implant surface have been described and developed. In vitro studies already have presented positive properties of hydrophilic surfaces on the mesenchymal stromal cells, osteoblast differentiation, and early matrix mineralization induction [13, 14]. According to Hamlet et al. [15], the hydrophilic surface of dental implants modulates the inflammatory response by inducing downregulation of inflammatory cytokines gene expression in monocytes. Besides, Hong et al. [16] affirmed that hydrophilic surfaces carry thrombogenic properties and improve bone healing. A clinical study verified that this kind of surface increases osteogenesis by osteogenic and angiogenic effects on gene expression [17].

In the process of osteoblast differentiation, several proteins of bone matrix are produced. Notably, bone sialoprotein (BSP) is a matrix protein that is a marker of osteogenesis [18]. Runt-related transcription factor 2 (RUNX2) upregulates the expression of bone matrix genes, including those for BSP. Furthermore, RUNX2 is a critical gene for both osteoblast differentiation and function [19].

Although several studies [3–5, 7, 11, 12, 14] have compared different implant surfaces and bone substitute materials, there is a lack of information regarding the behavior of those surfaces in the presence of bone substitute materials and in different periods. The purpose of this preclinical study was to evaluate, in a rabbit model, through immunohistochemical and molecular analysis, the cellular responses around titanium implants with different surface treatments installed in previously created defects filled or not with a bone substitute material. Moreover, to investigate the veracity of the hypothesis that porous-hydrophilic surface induces a faster bone formation when compared to porous surface.

## Materials and methods

This study was submitted and approved by the Ethics Committee in the Use of Animals of the São Paulo State University (Unesp), School of Dentistry, Araçatuba, Brazil (Protocol number FOA: 465-2016). In addition, this study strictly followed the ARRIVE Guidelines. Twenty-six *Oryctolagus cuniculus* male rabbits, weighting between 300 and 400 g and aged approximately 5 months, were selected. The animals were kept in separate cages with rabbit chow and water ad libitum.

A defect was created on both tibias of each animal. After that, an implant was installed in each defect. Finally, half of defects were filled with bone substitute material. Four groups were created:

- *BC*-*N:* porous surface implant (Neoporos surface, Neodent®, Curitiba, Brazil) and defect filled with blood clot
- *BC*-*A:* porous-hydrophilic surface implant (Acqua surface, Neodent®, Curitiba, Brazil) and defect filled with blood clot
- *HA*/*TCP*-*N*: porous surface implant (Neoporos surface, Neodent®, Curitiba, Brazil) and defect filled with biphasic ceramic composed of hydroxyapatite/β-tricalcium-phosphate (Straumann® Bone Ceramic, Straumann AG, Basel, Switzerland)
- *HA*/*TCP*-*A*: porous-hydrophilic surface implant (Acqua surface, Neodent®, Curitiba, Brazil) and defect filled with biphasic ceramic composed of hydroxyapatite/β-tricalcium-phosphate (Straumann® Bone Ceramic, Straumann AG, Basel, Switzerland).

Porous surface implants present cavities created through the concept of grit blasting followed by acid etching forming roughness of 2.5 and 5.0 μm on average. Afterwards, these roughnesses are standardized with the acid conditioning technique. These implants may be considered as hydrophobic (Neoporos surface, Neodent®, Curitiba, Brazil).

Porous-hydrophilic surface implant also present roughness created by the same concept previously described (grit blasting and acid etching). In addition, the hydrophilicity of this surface is obtained through a physical-chemical activation changing the negative charges of the surface to positive, without modifying the surface roughness as well as its topography (Acqua surface, Neodent®, Curitiba, Brazil).

**Fig. 1:**
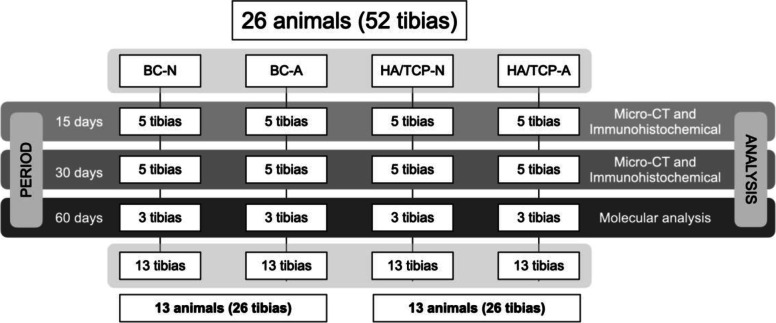
Materials and methods scheme (flowchart)

Twelve animals were euthanatized after 15 and 30 days of the surgical procedure. These samples were designated to micro-CT and immunohistochemical analysis. Six animals were euthanatized after 60 days of the surgical procedure and the samples were designated to molecular analysis (Materials and Methods Scheme) (Fig. 1).

### Surgical procedure

After 8 h fasting, the animals were sedated and anesthetized via intramuscular injection of a combination of 50 mg/ml of ketamine (Fracontar, Vibrac do Brasil Ltda., São Paulo, Brazil) and 5 mg/ml of xylazine (Rompum, Bayer AS, Saúde Animal, São Paulo, Brazil). Subsequently, the animals were submitted to trichotomy of the internal region of the left and right hind legs and antisepsis was performed. Local anesthesia was also applied with 0.3 ml/kg of 2% mepivacaine with 1:100.000 epinephrine (Mepiadre—Nova DFL, Rio de Janeiro, Brazil), for analgesia and hemostasis.

A 30-mm incision was done in planes over the tuberosity of both tibias. After delicate dissection of soft tissues and exposition of the bone, an osteotomy was created with an 8-mm trephine drill (Neodent®, Curitiba, Brazil), mounted in a 20:1 low-speed driller (SG20, Neodent®, Curitiba, Brazil) with the aid of an electric motor (NeoSurg XT Plus, Neodent®, Curitiba, Brazil) set to 800 rpm, creating a defect of 8-mm in length. The internal cortical was removed, preserving the external cortical of each tibia. After that, the drilling for implant installation was performed in the defect center initiating with the spear drill, followed by 2.0 mm, 2.8 mm, and 3.0 mm helical drills (Neodent®, Curitiba, Brazil), to accommodate an 8-mm-long titanium implant of 3.5 mm diameter (Drive CM Neoporos and Acqua surfaces, Neodent®, Curitiba, Brazil). Bone preparation was done under abundant irrigation with sterile saline. The implants were installed achieving primary stability in the external cortical of the tibia and the bone defect was then filled with the bone substitute or with blood clot.

The tissue was repositioned and sutured by planes. The muscle was closed with resorbable sutures (5-0 Vicryl Ethicon, Johnson & Johnson, São José dos Campos, Brazil) and the skin was closed with silk thread (4-0 Ethicon, Johnson & Johnson, São José dos Campos, Brazil) obtaining primary closure of the wound. The animals received a single dose of pentabiotic (0.1 ml/kg, Fort Dodge Saúde Animal Ltda., Campinas, Brazil) and dipyrone (1 mg/kg, Ariston Indústria Químicas e Farmacêuticas Ltda., São Paulo, Brazil).

After 15 and 30 days of surgical procedure, twenty animals were submitted to euthanasia by an overdose of anesthetic. Then, the tibias were removed and maintained and fixed in 10% formalin solution for 48 h, washed in running tap water for 24 h, and later stored in 70° ethanol. After 60 days, the last six animals were sedated and anesthetized for removal of the entire tibia while still alive. These specimens were maintained in liquid nitrogen and designated for molecular biology analysis. After that, the animals were submitted to euthanasia by an overdose of anesthetic solution.

### Micro-CT

A micro-CT (μCT) scanner (SkyScan 1176 Bruker MicroCT, Aatselaar, Belgium) was used to scan the samples, with the following specifications: camera pixel, 12.45; X-ray tube potency, 50 kilovoltage peak (kVp); X-ray intensity, 500 μA; integration time, 300 ms; filter, 1 mm aluminum (Al); voxel size, 9 μm^3^. All obtained images were filed and prepared for analysis using a specific software (NRecon, Data Viewer, CTAnalyser, Aatselaar, Belgium). Three imaging planes were evaluated: coronal, transaxial, and sagittal. A region of interest (ROI) was determined for each plane. For the coronal plane: 0.5 mm circular region around the entire diameter of the implant; for the transaxial and sagittal planes: a rectangular shape vertically from the top of the implant to the end of the second big thread, and 0.5 mm horizontally from the border of the implant. The threshold used in the analysis was 25–90 in grayscale and the mineralized tissue around the implants was assessed using four parameters: trabecular number (Tb.N), trabecular separation (Tb.Sp), trabecular thickness (Tb.Th), and connectivity density (Conn.Dn).

### Immunohistochemical analysis

Immunohistochemical analysis was performed to identify and localize the expression of proteins related to bone remodeling: run-related transcription factor 2 (RUNX2), osteopontin (OPN), osteocalcin (OCN), osteoprotegerin (OPG), receptor activator of nuclear factor kappa B ligand (RANKL). After micro-CT scanning, the samples were removed from 70° ethanol and washed in running tap water and decalcified in 10% ethylenediamine tetra-acetic acid (EDTA) solution for 90 days, changing the solution every 72 h. As soon as the decalcification was verified as completed, the implants were removed from the specimens. After that, samples were embedded in paraffin, sliced to a thickness of 6 μm, and placed on silanized slides. The immunolabeling protocols starts with procedures for deparaffinization and rehydration of the slides. Following, the slides were subjected to blockade of endogenous peroxidase by the application of 3% hydrogen peroxide for 30 min. The antigen retrieval was achieved by citrate phosphate buffer in humid heat. Unspecific link blockade was performed by 1% albumin bovine serum and skim milk. Subsequently, the primary antibody incubation was performed against the following proteins: RUNX2, OPN, OCN, OPG, and RANKL (Santa Cruz Biotechnology, Dallas, Texas, USA). Negative control was performed through the serum of primary antibody host. Secondary antibody used was the biotinilated donkey anti mouse (Jackson Immunoresearch Laboratories). Avidin Biotin was used in order to amplify the immunolabeling signal and diaminobenzidine (Dako, Glostrup, Denmark) was the cromogen. The samples were counterstained with Mayer Hematoxylin solution for visualization of the cell nuclei. The images were obtained using a camera coupled to a light microscope (Leica Reichert Diastar Prodcuts & Jung, Wetzlar, Germany). The expression levels of the proteins in the area close to the bone substitute material and implants were assessed through a protein labeling extension area: (−) without labeling (0% of cells/matrix); (+) mild/discrete labeling (< 25% of cells/matrix); (++) moderate labeling (25–50% of cells/matrix); (+++) strong labeling (< 50 of cells/matrix) [20]. A experienced examiner performed the descriptive analysis.

### Molecular analysis

At the 60-day period after implant installation, three samples from each group were submitted to explantation by a counter-torque wrench and the bone tissue adhered to the implants was collected for molecular biology experiments.

The reverse transcription polymerase chain reaction (RT-PCR) was performed with the objective of evaluating the gene expression of different markers in a certain stage of osteoblastic cells: Runx2 and BSP. The idea was to evaluate the expression of genes involved in osteoblast’s differentiation and consequently, the process of bone remodeling in late periods of periimplantar bone repair. For this purpose, primers designed by Primer Express 2.0 (Applied Biosystems, Foster City, CA, USA) were used.

Each bone fragment was carefully washed in PBS solution and subsequently frozen in liquid nitrogen so that the total RNA was isolated with the aid of the Trizol reagent (Life Technologies: Invitrogen, Carlsbad, CA, USA). RNA was quantified on a NanoDrop® 2000 spectrophotometer (Thermo Scientific NanoDrop Technologies, Wilmington, DE) for analysis of RNA integrity, purity, and concentration. Afterwards, the cDNA was made using 1 μg of RNA by the reverse transcriptase reaction (M-MLV: reverse transcriptase: Promega Corporation, Madison, Wisconsin, USA). The sequence of the primers was amplified and their products were electrophoresed with 1.5% agarose gel stained with ethidium bromide and visualized using Quantity One software (Bio-Rad Laboratories, Philadelphia, PA, USA).

Finally, RT-PCR was conducted using the StepOne system (Applied Biosystems, Foster City, CA, USA) and the SybrGreen (Primer Design Ltd, Southampton, UK) fluorescent dye system under the following cyclic conditions: 95° for 10 min for primary denaturation, 35 cycles of 95° for 15 s for tape opening, and finally 60° for 60 s for annealing of the primers and extension of the fragments. The relative gene expression was calculated by reference to the expression of the mitochondrial ribosomal proteins and normalized by the gene expression of the bone fragments removed from the implants in the given period of 60 days [21, 22].

### Statistical analysis

Collected data were tabulated using Microsoft Excel and submitted to statistical analysis. Data were submitted to Shapiro-Wilk test (normality) and Levene and Brown Forsythe tests (homogeneity of variances) and ANOVA two-way (*p* < 0.05). Residual analysis was also performed and in cases that effects were observed, the Tukey post-test was applied to identify where differences between groups occurred. The confidence interval was 95%. The software used were R 3.6.0 version and RStudio 1.2.1144 version.

## Results

### Micro-CT

#### Trabecular number (Tb.N)

**Fig. 2:**
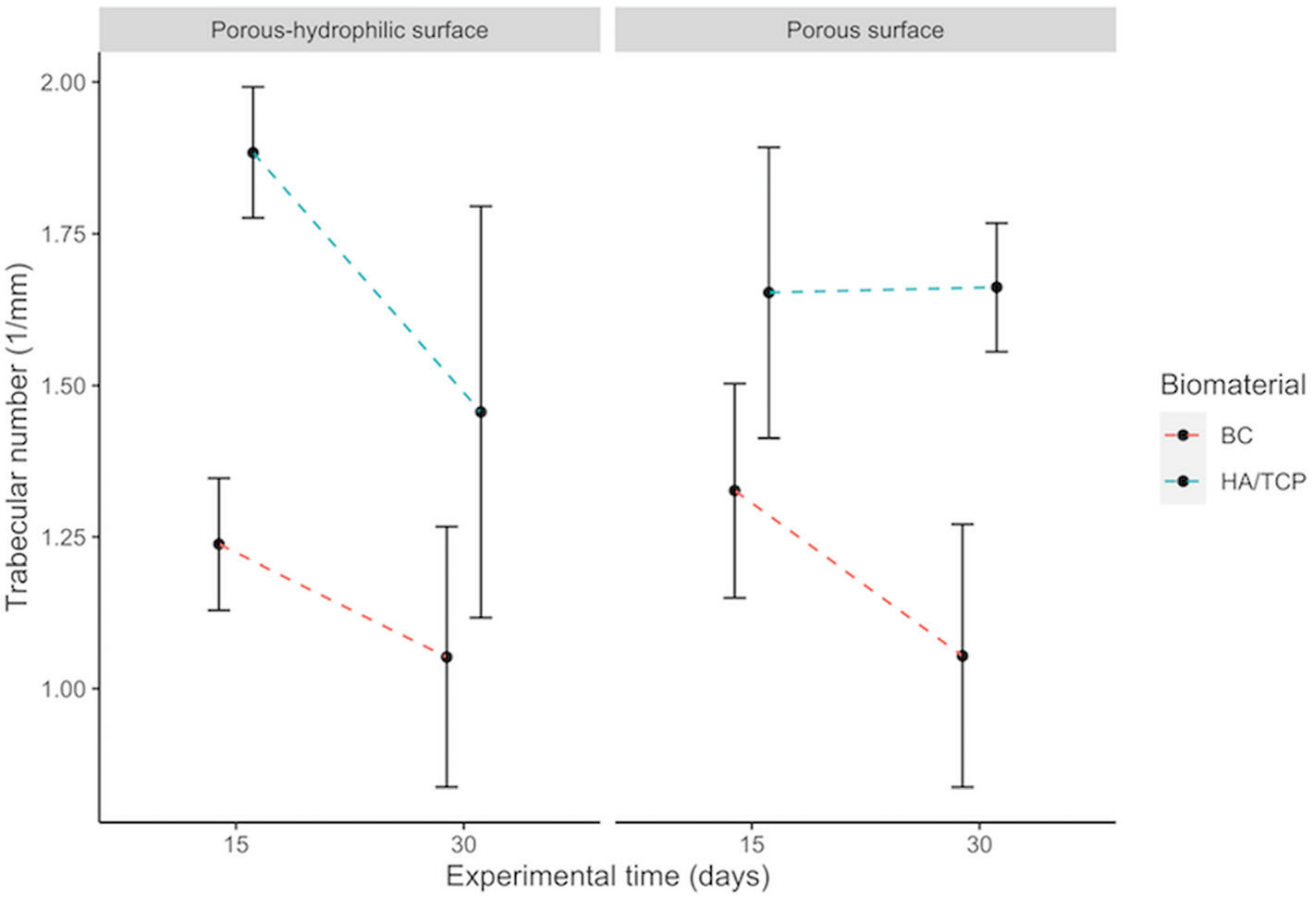
Interactions among implant surfaces, bone substitute material or blood clot and experimental period on trabecular number. Error bars represent the standard deviation around the means

There is a significant effect of the interaction between the presence bone substitute material or blood clot and the experimental period for the trabecular number (*p* = 0.001559). This means that the presence of the bone substitute material and the period of bone formation have their influence over the quantity of trabeculae, independently the implant surface. Table 1 shows the effect of the interaction between bone substitute material presence and period. Figure 2 complements the information about that interaction.

**Table 1:** Multiple comparisons of trabecular number

Defect filling × Experimental period	Mean differences	*p* value
HA/TCP:15-BC:15	0.48610	***< 0.001***
BC:30-BC:15	− 0.22915	0.14657
HA/TCP:30-BC:15	0.27665	**0.04507**
BC:30-HA/TCP:15	− 0.71524	***< 0.001***
HA/TCP:30-HA/TCP:15	− 0.20944	0.22277
HA/TCP:30-BC:30	0.50580	***< 0.001***

#### Trabecular separation (Tb.Sp)

**Fig. 3:**
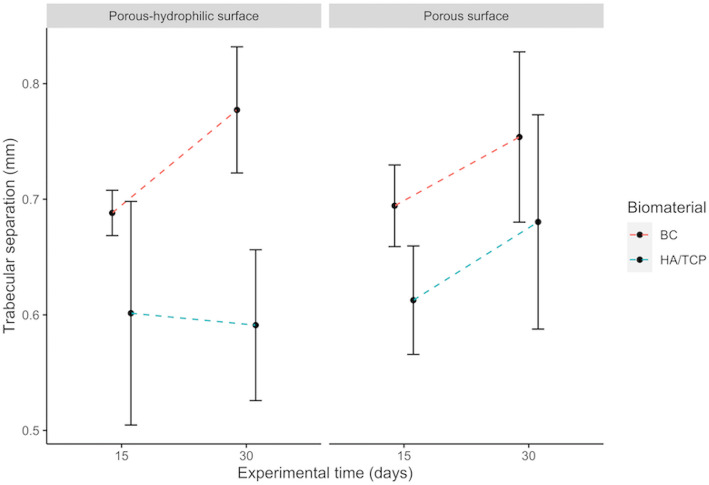
Interactions among implant surfaces, bone substitute material or blood clot and experimental period on trabecular separation. Error bars represent the standard deviation around the means

There is a significant effect of the interaction between the presence of bone substitute material or blood clot and the experimental period (*p* = 0.00731). Table 2 shows that the trabeculae present separation average mainly due to the presence of blood clot after 15 and 30 days, when compared to the presence of the bone substitute material. Separation between the trabeculae is smaller with bone substitute material after 15 days (Fig. 3).

**Table 2:** Multiple comparison of trabecular separation

Defect filling × Experimental period	Mean differences	*p* value
HA/TCP:15-BC:15	− 0.08421	0.14311
BC:30-BC:15	0.07431	0.25210
HA/TCP:30-BC:15	− 0.05552	0.56954
BC:30-HA/TCP:15	0.15852	***< 0.001***
HA/TCP:30-HA/TCP:15	0.02868	0.95560
HA/TCP:30-BC:30	− 0.12984	**0.00430**

#### Trabecular thickness (Tb.Th)

**Fig. 4:**
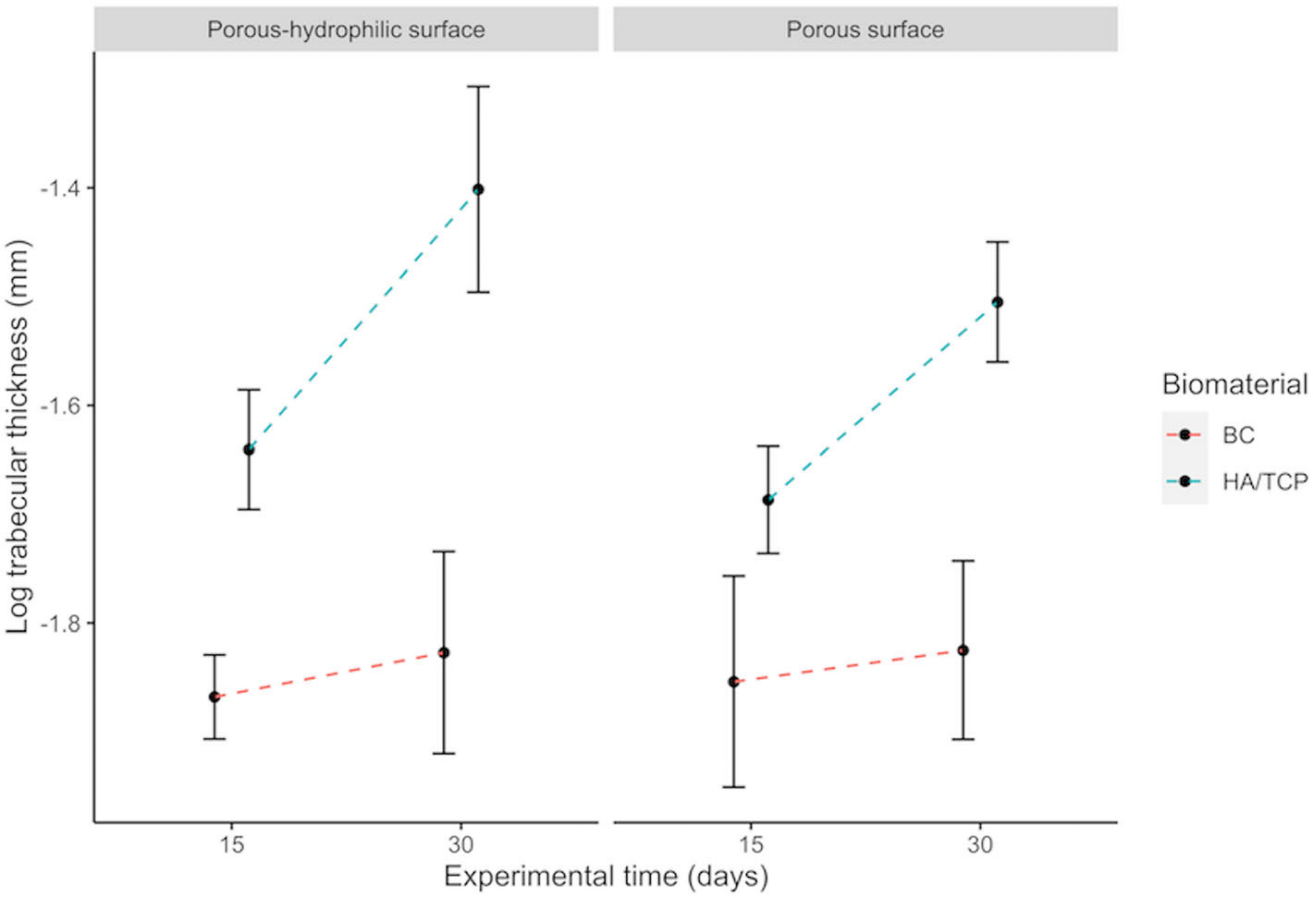
Interactions among implant surfaces, bone substitute material or blood clot and experimental period on trabecular thickness. Error bars represent the standard deviation around the means

There is a significant effect of the interaction between the presence of bone substitute material or blood clot and the experimental period (*p* = 0.010). Table 3, associated to Fig. 4, shows that the use of bone substitute material increases the trabecula thickness values, when compared to blood clot in the same experimental period. In the case of the bone substitute, trabecular thickness increases with time.

**Table 3:** Multiple comparisons of trabecular thickness (log)

Defect filling x Experimental period	Mean differences (log)	*p* value
HA/TCP:15-BC:15	0.197130390	***0***.***02356***
BC:30-BC:15	0.034846390	0.99212
HA/TCP:30-BC:15	0.407547270	**<** ***0***.***001***
BC:30-HA/TCP:15	− 0.162284000	0.09709
HA/TCP:30-HA/TCP:15	0.210416880	**0**.**01293**
HA/TCP:30-BC:30	0.372700880	< ***0.001***

One of the groups did not present normal distribution (porous surface implant, with bone substitute material and period of 15 days) with *p* = 0.04419 (Shapiro-Wilk test). The homoscedasticity assumption was also violated (*p* = 0.02777, Brown-Forsythe test). Then, it was opted for the transformation of trabeculae thickness data into logarithm. The groups showed homoscedasticity after the transformation (*p* = 0.05172, Brown-Forsythe test).

#### Connectivity density (Conn.Dn)

**Fig. 5:**
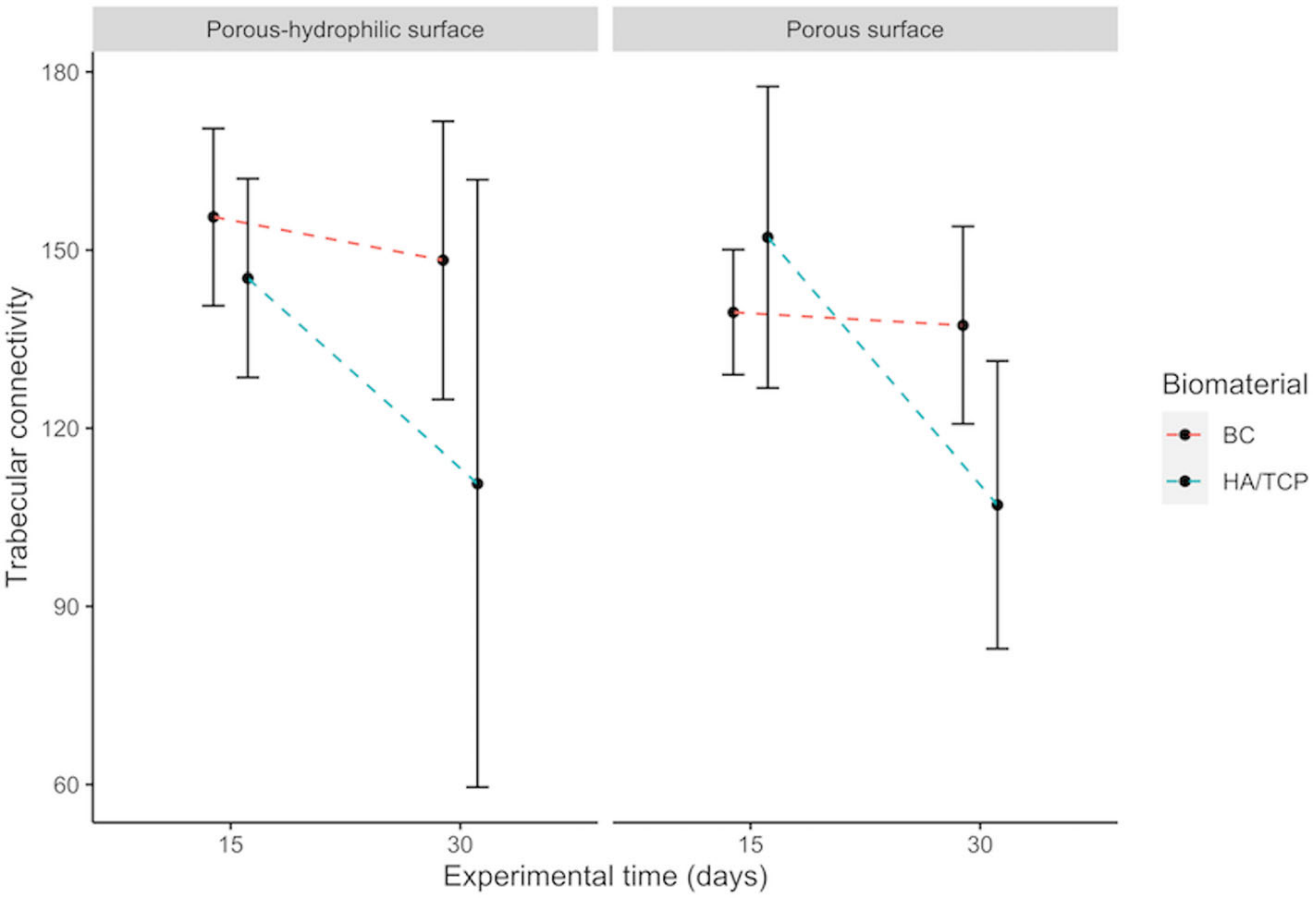
Interactions among implant surfaces, bone substitute material or blood clot and experimental period on connectivity density. Error bars represent the standard deviation around the means

There is no significant effect of the interaction between implant surface, presence of bone substitute material or blood clot, and experimental period, nor effect of each of the variables in isolation for this parameter, even if the graphic (Fig. 5) apparently demonstrated that implants in the presence of blood clot generally have a greater connectivity density.

### Immunohistochemical analysis

**Fig. 6:**
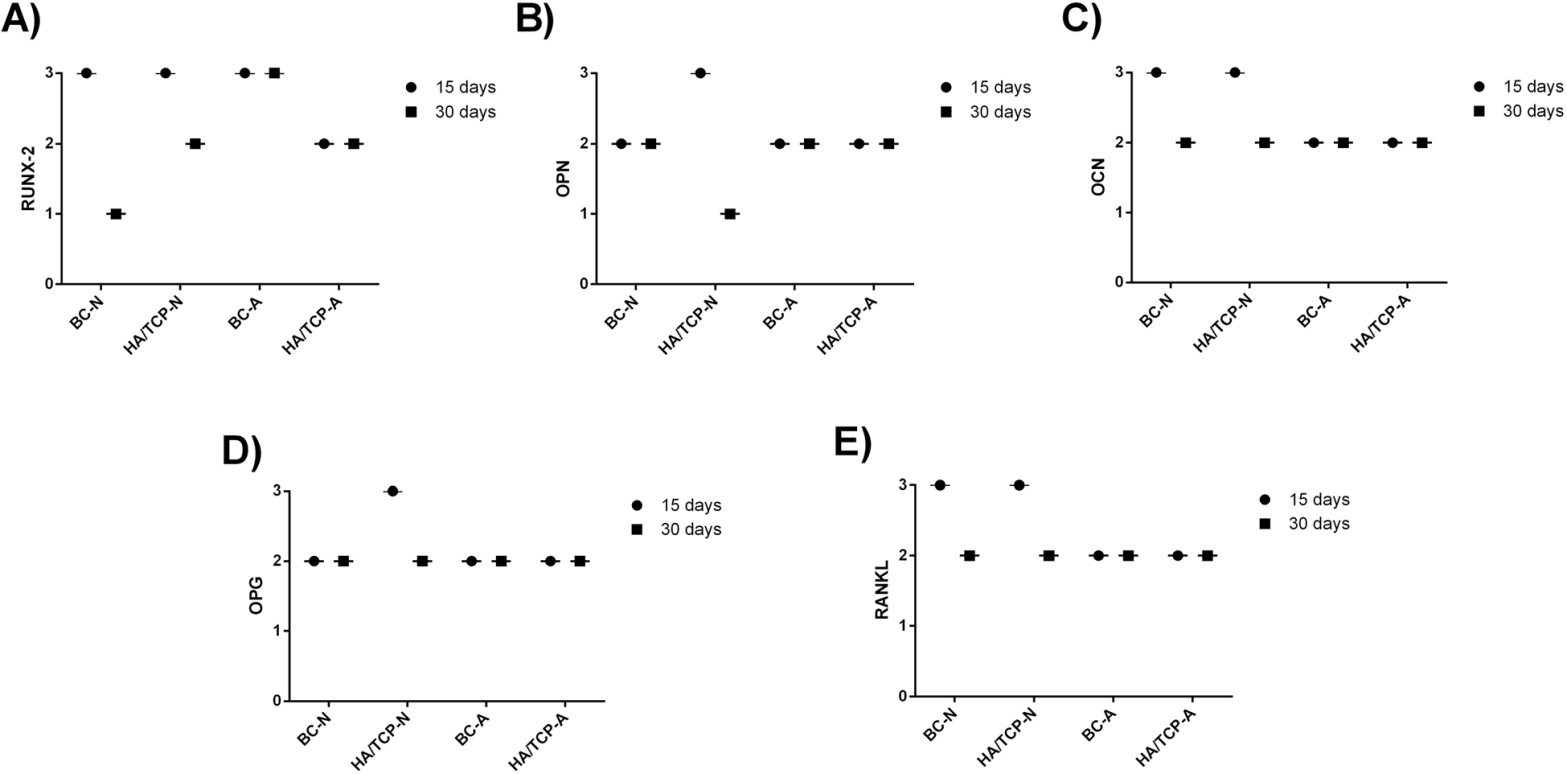
Representative graphic of immunolabeling of all antibodies. **a** RUNX2. **b** OPN. **c** OCN. **d** OPG. **e** RANKL

**Fig. 7:**
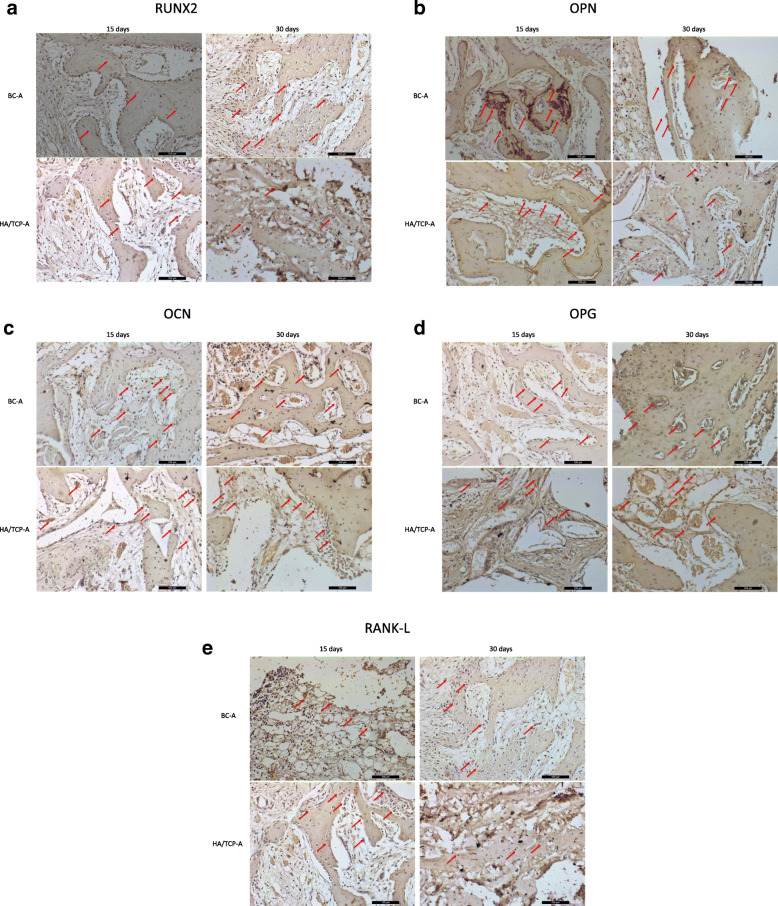
Labeled samples for all antibodies according to porous-hydrophilic surfaces groups at both periods (**a**–**e**). Immunolabeling of proteins (red arrow)

**Fig. 8:**
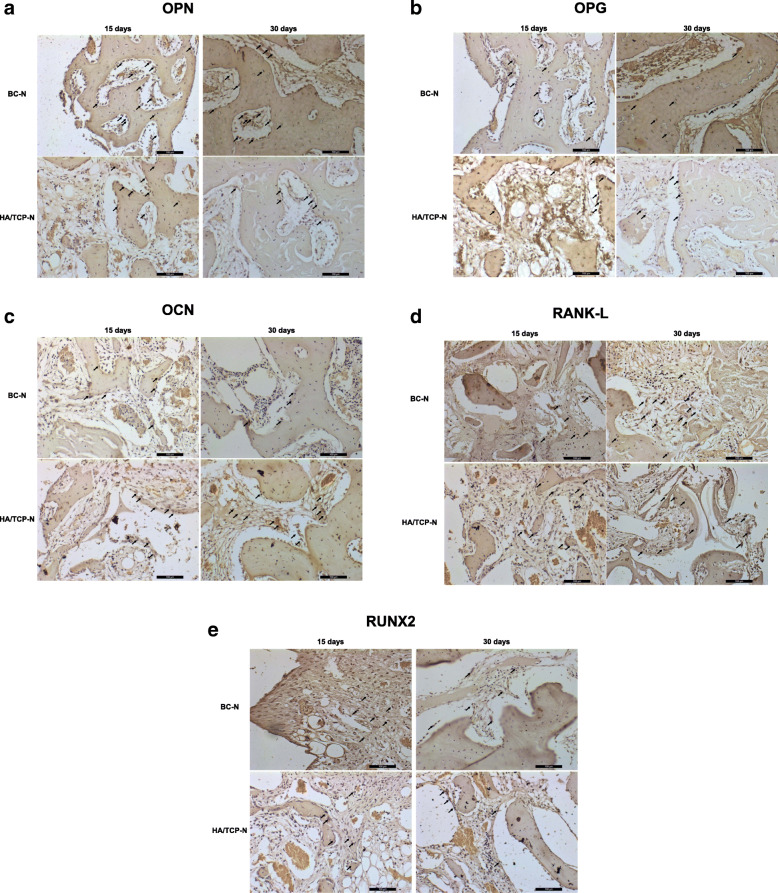
Labeled samples for all antibodies according to porous surfaces groups at both periods (**a**–**e**). Immunolabeling of proteins (black arrow)

The indirect immunoperoxidase technique was used with the diaminobenzidine as chromogen, whose brownish staining labeled the cells and extracellular matrix positive for the antigens evaluated, close to bone substitute material and implants, in order to characterize the cells activity in the region of interest (periimplantar bone). During the description of the results, the labeling was categorized as mild/discrete (+), moderate (++), or intense (+++) according to the area/quantity of positive cells for each antigen (Table 4 and Fig. 6). Figures 7 and 8 show the labeled samples for all antibodies, according to all groups, at both periods.

**Table 4:** Representative scores (most frequently observed) of the immunolabeling, categorized as mild/discrete (+), moderate (++), or intense (+++), according to the area/quantity of positive cells for each antigen in all groups in both periods

	RUNX2	OPN	OCN	OPG	RANKL
15 days					
BC-N	+++	++	+++	++	+++
HA/TCP-N	+++	+++	+++	+++	+++
BC-A	+++	++	++	++	++
HA/TCP-A	++	++	++	++	++
30 days					
BC-N	+	++	++	++	++
HA/TCP-N	++	+	++	++	++
BC-A	+++	++	++	++	++
HA/TCP-A	++	++	++	++	++

#### Porous surface groups

##### RUNX2

At 15 days, the presence of immature osteoblasts in intense proliferation and differentiation process is observed in both groups. There is presence of extracellular matrix around cells intensely labeled throughout the region of interest. At 30 days, a reduction of the presence of RUNX-2-positively labeled cells is observed in the BC-N group, as expected at this stage. Thus, the labeling for RUNX-2 is considered mild. In the HA/TCP-N group, more RUNX-2-labeled cells are observed and there is a delay in the development of repair bone tissue. Thus, immunolabeling for RUNX-2 is categorized as moderate.

##### OPN

In the BC-N groups, labeling is moderate for the presence of osteoblasts, but the repair process is well developed in both periods. In HA/TCP-N groups, at 15 days, an intense labeling for this protein is observed on osteoblasts around the new bone tissue formation, as well as positive labeling on cells located around the non-mineralized extracellular matrix. However, despite the intense immunostaining for this protein, the repair stage is delayed compared to the BC-N group at 15 days. At 30 days, it is observed that the bone repair process has evolved and a slight labeling for osteoblasts around the newly formed bone tissue is observed.

##### OCN

In the BC-N groups, intense labeling is observed in osteoblasts located around the newly formed bone tissue, as well as in cells located in the non-mineralized extracellular matrix at 15 days. At 30 days, there is important labeling, considered moderate, for osteocytes trapped within the newly formed bone tissue, characterizing its maturity. In the HA/TCP-N groups, at 15 days, the immunostaining showed similar results to those observed in the BC-N group in the same period. At 30 days, there is a delay in the repair stage, mainly evidenced by the moderate osteocalcin labeling in cells located in the non-mineralized extracellular matrix. There is a slight labeling for osteocytes trapped in the formed bone tissue, in smaller quantity when compared to the BC-N group at the same period.

##### OPG

In the BC-N groups, labeling is moderate at both periods, being positively labeled in osteoblasts present around the newly formed bone tissue. In the HA/TCP-N groups, at 15 days, there is an intense positive labeling for this protein; however, most of the labeled cells are located in the non-mineralized extracellular matrix, which characterizes the delay of the repair stage when compared to the BC-N group at 15 days. At 30 days, a moderate labeling for osteoprotegerin is observed, with remnant of non-mineralized extracellular matrix, once again showing a delay in the repair stage when compared to the BC-N group for the same period.

##### RANKL

IN BC-N groups, RANKL labeling is intense at 15 days and labeled cells are observed next to the non-mineralized extracellular matrix. At 30 days, moderate labeling is observed for the cells located near the newly formed bone. In the HA/TCP-N groups, intense RANKL labeling is observed at 15 days in cells located in the non-mineralized extracellular matrix. At 30 days, moderate labeling is seen in cells located near the non-mineralized extracellular matrix. It is noteworthy that the repair stage is delayed at both 15 and 30 days of the HA/TCP-N groups compared to the BC-N groups.

The associated evaluation of OPG and RANKL shows a balance between the bone formation and resorption stages, represented by OPG and RANKL respectively, in both BC-N and HA/TCP-N groups.

#### Porous-hydrophilic surface groups

##### RUNX2

At 15 days, the BC-A group shows an intense positive labeling for RUNX2, observed in cells surrounded by the non-mineralized extracellular matrix. The organization of bone tissue is noted, still incipient, near the negative area of the implant thread. At 30 days, in this same group, it is still possible to observe an intense positive labeling for this transcription factor in many regions of the evaluated area of interest and also areas of bone formation and immature osteoblasts are found near the newly mineralized bone matrix. In the HA/TCP-A groups, at 15 days, it is possible to observe moderate positive labeling for pre-osteoblasts and immature osteoblasts. The repair stage is advanced when compared to the BC-A group at 15 days. At 30 days, moderate positive RUNX-2 labeling is observed for pre-osteoblasts and immature osteoblasts in a formed and organized bone tissue.

##### OPN

In the BC-A groups, at 15 days, the OPN labeling is moderate for osteoblasts. However, the presence of intense labeling for this protein in cementing lines in the bone formed near the implant thread is highlighted. Moreover, positive labeling is also observed for osteoblasts located near the newly formed bone. At 30 days, there is moderate labeling in osteoblasts located near the newly formed bone. In the HA/TCP-A groups, moderate osteopontin labeling is observed in cells located near the non-mineralized extracellular matrix at both periods.

##### OCN

In BC-A groups, at 15 days, the OCN labeling is moderate. Its presence is evidenced in cells located in the non-mineralized extracellular matrix and also in osteocytes trapped in newly formed bone. At 30 days, moderate osteocalcin labeling is also observed in osteocytes trapped in the newly formed bone, showing a higher degree of maturity in the repair stage when compared to the previous period. In the HA/TCP-A groups, moderate osteocalcin labeling is seen at 15 days. At 30 days, moderate immunostaining for osteocalcin is evidenced, highlighting the presence of many cells positive for this protein located in the non-mineralized extracellular matrix, characterizing a delay in the repair process when compared to group BC-A at the same period.

##### OPG

In BC-A groups, at 15 days, the labeling is moderate, especially in few osteoblasts and in cells located in the non-mineralized extracellular matrix. At 30 days, it is possible to observe moderate osteocyte labeling for this protein, characterizing the degree of maturity of the newly formed bone. In the HA/TCP-A groups, at 15 days, moderate labeling is observed in cells located in the non-mineralized extracellular matrix, especially around fragments of the bone substitute material. At 30 days, moderate labeling is seen in cells located in the non-mineralized extracellular matrix, highlighting the presence of large amount of blood vessels.

##### RANKL

In the BC-A groups, at 15 days, the labeling is moderate in cells located in the non-mineralized extracellular matrix. It remained moderate in osteoblasts near the newly formed bone after 30 days. For the HA/TCP-A groups, moderate RANKL labeling is observed in osteocytes surrounded by newly formed bone, at 15 days. After 30 days, moderate labeling in osteoblasts and osteocytes close to bone tissue is observed.

Again, the associated evaluation of OPG and RANKL shows a balance between bone formation and resorption stages, in both BC-A and HA/TCP-A groups.

### Molecular analysis

**Fig. 9:**
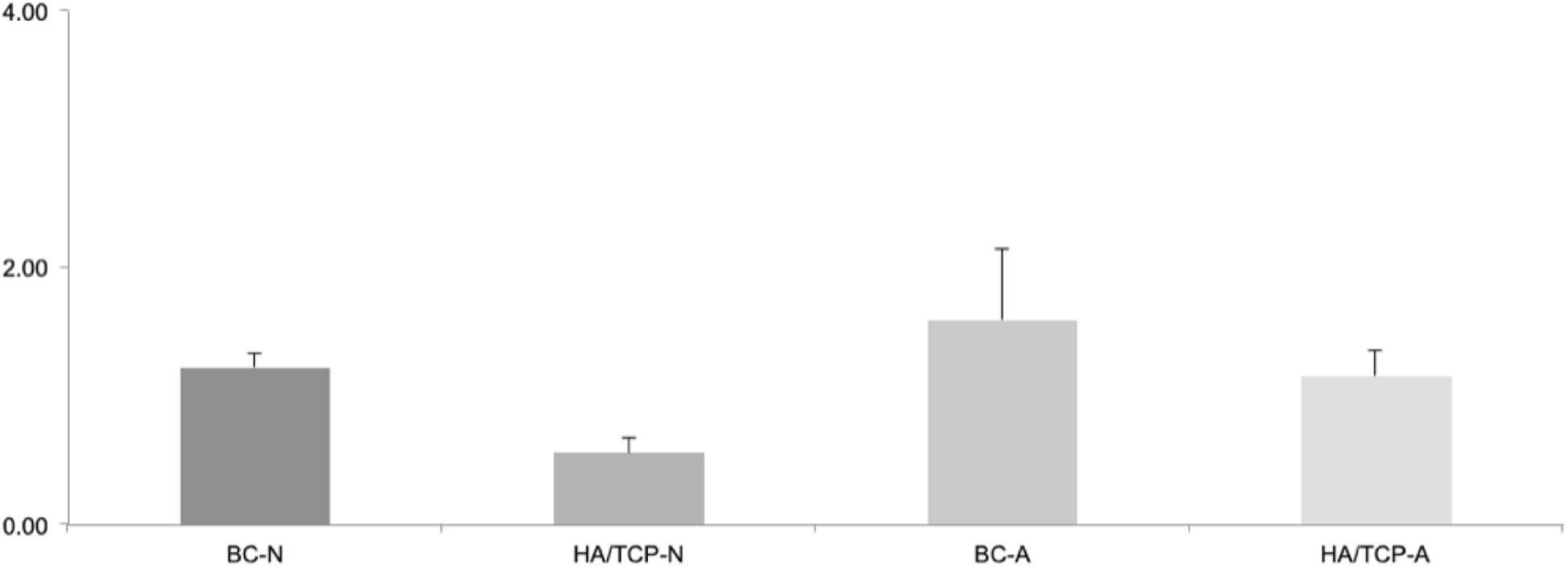
RUNX2 relative expression

**Fig. 10:**
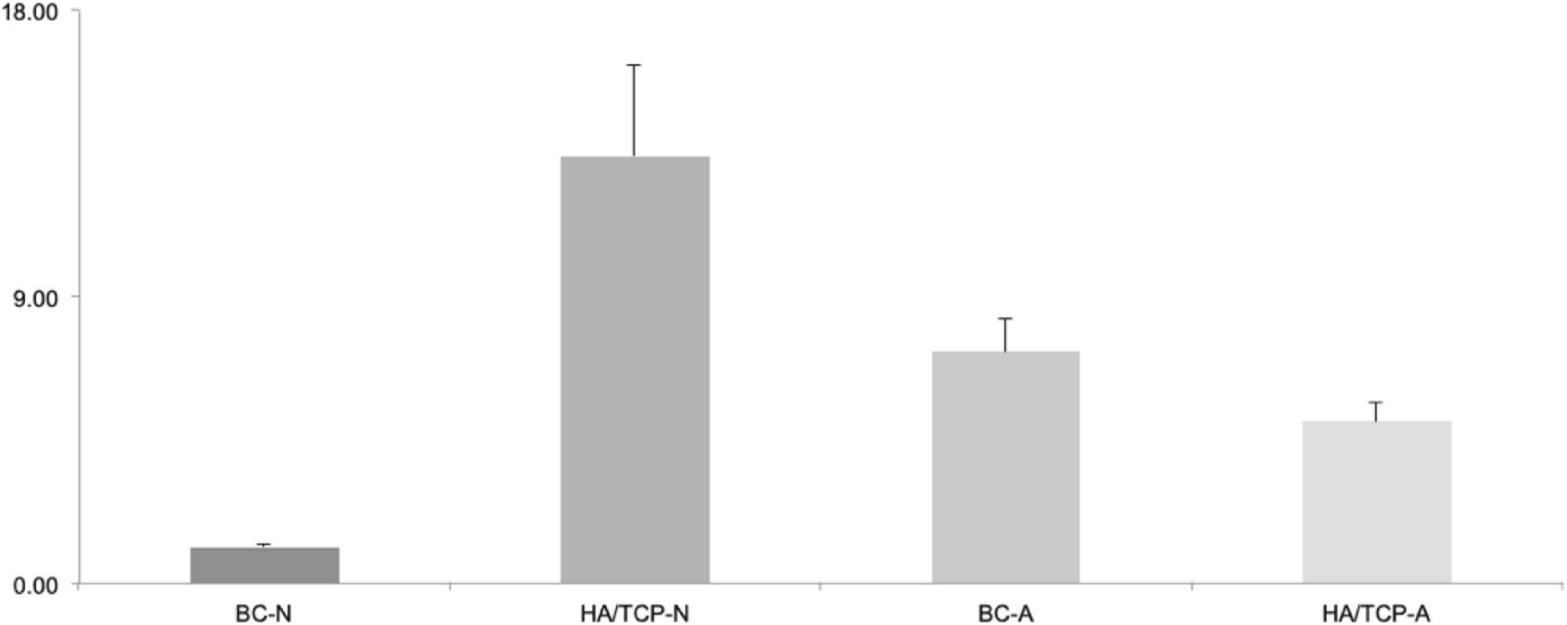
BSP relative expression.

Twelve samples were submitted to RT-PCR to verify the expression of two different genes: Runx2 and BSP. Figure 9 represents the relative expression of the Runx2 gene. Figure 10 represents the relative expression of the BSP gene.

The expressions of the other groups were evaluated in relation to the BC-N group (control). For this reason, the control group always presents an expression average value close to 1. The average value presented by the other groups represents how many times the expression of the evaluated gene is greater in relation to the control group.

Analyzing the graphics, it is possible to observe that Runx2 gene expression is better in the groups of porous-hydrophilic surface implants. The comparison between the groups HA/TCP-N and BC-A resulted in *p* < 0.0001, that is, a very significant difference between them. Furthermore, the comparison between HA/TCP-N and HA/TCP-A resulted in *p* = 0.0361.

In relation to BSP gene expression, as well as that of the Runx2 gene, both are better in the groups of porous and hydrophilic surface implants. When compared to the control group, the group without bone substitute material resulted in a *p* = 0.0025, that is, a very significant difference, and the bone substitute material group resulted in a *p* = 0.0289. However, the HA/TCP-N group has a better expression of the BSP gene when compared to all the other groups, resulting in very significant differences in relation to the BC-A group and to the BC-N and HA/TCP-A groups: *p* < 0.0001 (BC-N), *p* = 0.0018 (BC-A), *p* < 0.0001 (HA/TCP-A).

In porous and hydrophilic surface implants, there is a better expression of the Runx2 gene. In addition, the bone substitute material does not appear to increase the expression of this gene when compared to blood clot groups. Still, within the bone material substitute groups, we observed a better expression of the gene in the group of porous-hydrophilic surface implants. Regarding the expression of the BSP gene, we can observe a better expression in the groups of porous-hydrophilic surface implants when compared to the control group (BC-N).

## Discussion

The better understanding of the behavior of biological cells and biomaterials, such as titanium implant surfaces, can provide important information for the development of new clinical techniques for patient rehabilitation.

Immunostaining was performed to evaluate the activity of proteins that play an important role in osteogenic activity in the periimplantar region, evaluating the positive labeling for these proteins, close to the bone substitute material and implant’s surface, from the osteoblast differentiation stage (RUNX2), to the synthesis of the organic matrix (OPN), mineralization (OCN), and finally bone remodeling (New Tumor Necrosis Factor Members: OPG/RANKL). This is an ordinary qualitative analysis, through score attribution. The information about protein expression in the area close to the implant surface is very important, especially when there is a biomaterial filling the periimplantar defect. The descriptive analysis showed similar findings among the experimental groups. Moreover, a more intense protein activity was observed for the shorter experimental period of 15 days, with diminishing activity after 30 days. These results suggest that porous-hydrophilic surfaces can accelerate the bone formation in defects filled with bone substitute material or blood clot with the activation of extracellular matrix proteins that have an important role in osteogenic activity. This is supported by several studies [13, 23, 24]. In contrast, the immunohistochemical results also suggest that the porous surface can provide similar results in similar conditions.

Several studies [13, 16, 17] demonstrated the potential of hydrophilic implant surfaces about the enhancement of relevant protein expression for bone tissue formation. These studies used different methodologies and periods from this study and the comparison among them remains somewhat incomparable. Unfortunately, the lack of studies with the same periods and cell analysis limited the discussion of this study.

Bone defect healing starts from cell proliferation allowed by a stable fibrin clot promoting formation of granulation tissue. This tissue forms new connective tissue with mature collagen that will promote the intramembranous ossification, leading to the bone defect to be filled by the newly formed bone. In this condition, the basic multicellular unit (BMU) is accurate. The BMU is a balance of bone formation and resorption, osteoblasts and osteoclasts [25, 26]. Following such concepts, during the defect healing, the newly formed bone must develop mature bone characteristics, increasing trabecular thickness and decreasing trabecular space. Microscopically, the trabecular thickness (Tb.Th) increased in this study, corroborating the information above. Regarding the trabecular separation (Tb.Sp), results are not in accordance to that concept. However, the porous-hydrophilic surface, in the presence of bone substitute material, strictly followed it. Moreover, by the immunohistochemical analysis, it is possible to affirm that this study rigidly follows the BMU concepts.

Studies already presented, by means of in vitro data, the positive influences of hydrophilic surfaces over the differentiation of osteoblasts [13, 14]. Besides, Hong et al. [16] stated that hydrophilic surfaces can improve bone healing. The wettability of hydrophilic surfaces favors initial phases of wound healing, leading to a faster osteogenesis [27]. In this study, according to the molecular analysis performed at 60 days postoperatively, when the remodeling step of the repair process is in evidence, porous-hydrophilic surfaces presented a higher expression of RUNX2. This seems to be a better indicative of osteogenesis, as well as an important factor on the pre-osteoblastic differentiation process. This expression was not influenced by the presence of the bone substitute material. Once the Runx2 induces the differentiation of osteoblasts, these results suggest that hydrophilic surfaces have an individual influence over the osteogenesis independently of the presence of bone substitute material.

In contrast, the BSP expression is higher for the association between porous surface and bone substitute material. Although BSP function remains unclear, it is an initial marker of osteoblasts and its ability to bind with hydroxyapatite crystals plays an important role in early stages of mineralization [28]. The results of this study suggest that the presence of bone substitute material can increase the BSP expression, but the porous-hydrophilic surface decreases its expression by favoring a faster bone formation. On the other hand, the expression in high levels of BSP on the extracellular matrix indicates mature or in formation bone suggesting that, after 60 days, HA/TCP-N presents more mature bone or more bone formation activity [29, 30].

## Conclusion

Taken all the information together, it is possible to state that porous-hydrophilic implant surfaces present similar behavior to porous implant surfaces in defects filled with blood clot or bone substitute material. However, some features demonstrate that the porous hydrophilic surface can improve and accelerate protein expression and bone formation. Finally, according to this study, the previously raised hypothesis has not sufficiently supporting to be accepted.

## Data Availability

All data generated or analyzed during this study are included in this published article (and its supplementary information files).

## Abbreviations

HA/TCP: Biphasic hydroxyapatite/β-tricalcium-phosphate
BC: Blood clot
RUNX2: Run-related transcription factor 2
OPN: Osteopontin
OCN: Osteocalcin
OPG: Osteoprotegerin
RANKL: Receptor activator of nuclear factor kappa B ligand
RT-PCR: Reverse transcription polymerase chain reaction
Micro-CT: Microtomographic
Tb.N: Trabecular number
Tb.Sp: Trabecular separation
Tb.Th: Trabecular thickness
Conn.Dn: Connectivity density
